# Neurons Induce Tiled Astrocytes with Branches That Avoid Each Other

**DOI:** 10.3390/ijms23084161

**Published:** 2022-04-09

**Authors:** Mariko Kato Hayashi, Kaoru Sato, Yuko Sekino

**Affiliations:** 1 Department of Food Science and Nutrition, Faculty of Food and Health Sciences, Showa Women’s University, Tokyo 154-8533, Japan; 2 Medical School, International University of Health and Welfare, Chiba 286-8686, Japan; 3 Laboratory of Neuropharmacology, Division of Pharmacology, National Institute of Health Sciences, Kawasaki 210-9501, Japan; kasato@nihs.go.jp; 4 Endowed Laboratory of Human Cell-Based Drug Discovery, Graduate School of Pharmaceutical Sciences, the University of Tokyo, Tokyo 113-0033, Japan; yukos@g.ecc.u-tokyo.ac.jp

**Keywords:** astrocyte, neuron, tiling, glutamate transporter

## Abstract

Neurons induce astrocyte branches that approach synapses. Each astrocyte tiles by expanding branches in an exclusive territory, with limited entries for the neighboring astrocyte branches. However, how astrocytes form exclusive territories is not known. For example, the extensive branching of astrocytes may sterically interfere with the penetration of other astrocyte branches. Alternatively, astrocyte branches may actively avoid each other or remove overlapped branches to establish a territory. Here, we show time-lapse imaging of the multi-order branching process of GFP-labeled astrocytes. Astrocyte branches grow in the direction where other astrocyte branches do not exist. Neurons that had just started to grow dendrites were able to induce astrocyte branching and tiling. Upon neuronal loss by glutamate excitotoxicity, astrocytes’ terminal processes retracted and more branches went over other branches. Our results indicate that neurons induce astrocyte branches and make them avoid each other.

## 1. Introduction

Astrocytes approach neurons with their finely branched processes to support neuronal functions. Astrocyte branches radiate from their soma with a star-like morphology and divide multiple times to form thousands of delicate terminal processes [1]. Neurons play critical roles in inducing the morphological and functional maturation of astrocytes. Astrocytes co-cultured with neurons form multiple branches, while astrocytes in monoculture are polygonal [2,3,4,5,6,7]. Neurons also induce glutamate transporter expression in astrocytes [8,9]. Glutamate transporters at astrocyte processes remove glutamate released to excitatory synapses [2,8,10]. They terminate the signal to prepare for the next glutamate release and protect neurons from excitotoxicity.

Each astrocyte develops a unique spatial domain with limited penetration of branches from neighboring astrocytes in the brain [1,11,12]. This non-overlapping territory formation is called tiling. Tiling is an excellent way to establish uniform coverage of the brain. However, how astrocytes regulate the growth of their branches to tile is not known. One possibility is that the multi-order spongiform branching of astrocyte processes sterically limits the penetration of processes from neighboring astrocytes. Another possibility is that astrocyte branches actively avoid neighboring branches. Alternatively, it is also possible that astrocytes initially extend overlapping branches and then retract or prune them to establish territories.

In the rat hippocampus, astrocytes extend overlapping branches during the first two weeks after birth when neurons form synapses. After that, astrocytes ramify extensively and develop clear boundaries within a month [13]. This report suggests that neuronal maturation, such as synapse formation, may be necessary for astrocyte tiling. However, how neurite extension or synapse formation contributes to astrocyte branching, tiling, and functional maturation is unknown.

Astrocytes tile in a healthy brain. In contrast, reactive astrocytes produced under seizure-related pathological conditions show reduced branching and do not tile [14]. Whether neuronal damage caused by glutamate excitotoxicity is sufficient for tiling loss or other factors such as microglia activation or leukocyte infiltration are required is not known.

In this study, we performed time-lapse imaging of neuron–astrocytes co-culture. EGFP-labeled astrocytes preferentially expand branches in directions without other branches. Furthermore, neurons that had just started to grow dendrites induced astrocyte branching and tiling. Upon glutamate application, neurons are damaged due to excitotoxicity, and astrocyte branches begin to explore the area and violate the tiling. Our results indicate that astrocyte tiling is a consequence of the specific avoidance of other astrocyte branches induced by neurons.

## 2. Results

### 2.1. Neurons Induce Astrocytes Branching and Tiling

**Figure 1:**
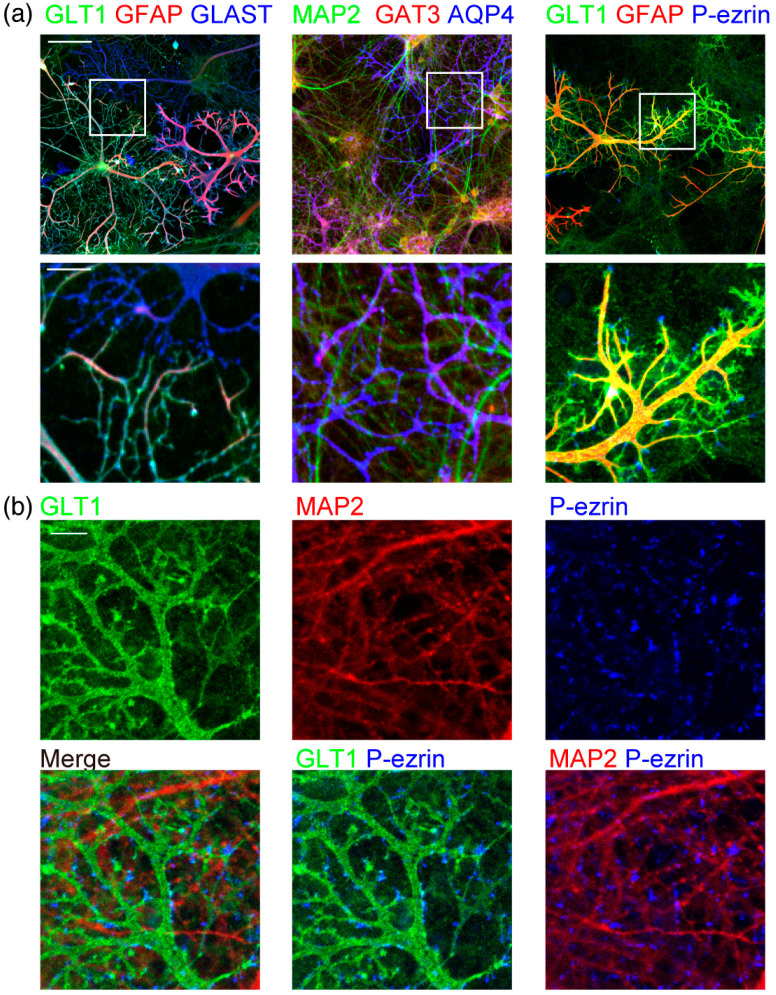
Morphology of astrocytes and neurons in co-culture. (**a**) Astrocytes and neurons in rat cerebrum neuron–astrocyte co-culture on DIV15, immunostained by neuronal or astrocytic marker antibodies. Left, astrocytic markers of GLT1 (green), GFAP (red), and GLAST (blue). Middle, a neuronal dendritic marker of MAP2 (green) and astrocytic markers of GAT3 (red) and AQP4 (blue). Right, GLT1 (green), GFAP (red), and phospho-ezrin (blue). Top, scale bar, 100 µm. Bottom, an enlarged view of the area indicated with a square in the left image. Scale bar, 25 µm. Similar results were obtained for 3–7 independently prepared cultures. Images of each channel are provided in Figure S1. (**b**) High-magnification images of astrocytes and neurons in rat cerebrum neuron–astrocyte co-culture on DIV19. Astrocytic marker GLT1 (green), MAP2 (red), and phospho-ezrin (blue). Scale bar, 10 µm. Similar results were obtained for 3 independently prepared cultures.

We prepared the primary neuron–astrocyte co-culture from the P1 (postnatal day 1) rat’s entire cerebral cortex. Astrocytes formed highly branched processes (Figure 1a) when cultured with 5 µM AraC (cytosine arabinoside) to restrict their proliferation from DIV1 (days in vitro 1). Astrocyte branches rarely extend over other astrocyte branches from the same or neighboring astrocytes. In contrast, MAP2 (microtubule-associated protein 2) positive neuronal dendrites extend over other dendrites and astrocyte branches. This result indicates that astrocyte branches stop expanding around other astrocyte branches. 

We immunostained the neuron–astrocyte co-culture with transporter proteins. The cellular location and expression of these major astrocytic markers are important for the functional maturation of astrocytes. Astrocytes express two glutamate transporters, GLAST (glutamate aspartate transporter) and GLT1 (glutamate transporter 1), for the clearance of glutamate released to synapses. Both GLAST and GLT1 are localized to the astrocyte plasma membrane, including their branches approaching neurons (Figure 1a). Astrocytic intermediate filament GFAP (glial fibrillary acidic protein) was in most major branches but did not reach their small glutamate transporter positive process tips. GABA (γ-aminobutyric acid) transporter GAT3 (GABA transporter 3) was also localized to astrocyte branches. Astrocytic water channel AQP4 (aquaporin 4) is localized to astrocyte endfeet facing the blood vessels in the brain [15]. AQP4 was uniformly localized to the plasma membrane in this culture without blood vessels. 

The peripheral astrocyte processes are the tips of astrocyte processes that approach neurons. It was reported that in astrocyte monoculture with fewer branches, phospho-ezrin is located at the tips of the branches, while GFAP is at the stem [16,17]. Astrocytes with multi-order branches had phospho-ezrin at the tips of the processes, where GFAP at the stem does not reach (Figure 1a). These phospho-ezrin positive processes are at the tips of GLT1 positive astrocyte processes. They are often located around neuronal dendrites (Figure 1b). Astrocytes in the brain have fine sub-micron diameter spongiform processes [1,11]. However, the astrocyte marker antibodies we used did not detect the spongiform structures in this culture.

### 2.2. Astrocyte Branching and Tiling Proceeds during Neuronal Dendritic Growth, before Synaptic Maturation

**Figure 2:**
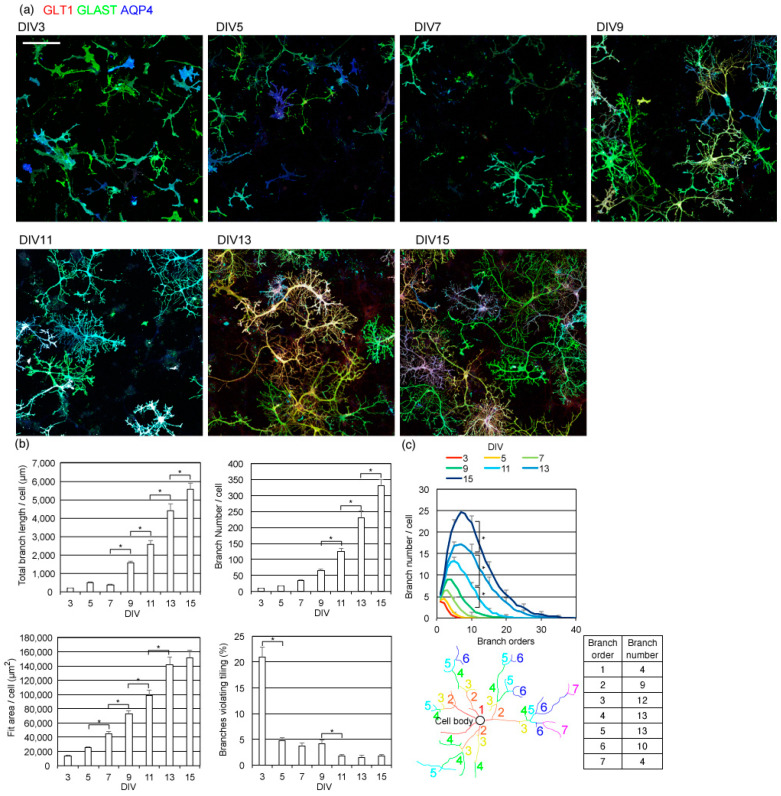
Time-course of astrocyte branch formation. (**a**) Astrocyte process formation in rat cerebrum primary neuron–astrocyte co-culture. Astrocytes were immunostained by GLT1 (green), GLAST (red), and AQP4 (blue). Scale bar, 200 µm. Similar results were obtained for 4 independently prepared cultures. Images of each channel are provided in Figure S2. (**b**) Top left, the average of the total length of branches on astrocytes. Top right, the average number of branches on each astrocyte. Bottom left, the average area covered by each astrocyte, estimated by the area of a rectangle. Bottom right, the average percentage of branches that violate the tiling. Asterisks, *p* < 0.05 two-day interval pairs by Tukey–Kramer test. (**c**) Topological branch order analysis of astrocytes. Top, results of topological branch order analysis for astrocytes on each DIV. Error bars are shown for every 5 branch orders. Asterisks, *p* < 0.05 two-day interval pairs by Tukey–Kramer test of order 10 branch numbers. Bottom, an example of a topological branch order analysis showing in color, showing how branches are ordered and counted. (**b**,**c**) Same sets of images were used. Mean ± s.e.m. of 88–208 cells. Similar results were obtained for 3 independently prepared cultures.

Next, we obtained time-course immunostained images of astrocytes to study which stage astrocyte tiling starts. Most astrocytes on DIV3 do not have thin multi-order branches yet (Figure 2). However, by DIV5, many astrocytes grew multi-order branches. Remarkably, astrocyte processes avoided processes from the same or other astrocytes already at DIV5, and each astrocyte formed discrete territories. Astrocytes kept expanding while maintaining the boundaries with other astrocytes, gradually forming multi-order branches. The total length and the number of branches per astrocyte kept growing from DIV5 to DIV15, indicating that astrocytes grow by forming new branches and expanding them (Figure 2b). Furthermore, the area covered by each astrocyte, estimated by a rectangle area that just covers it, also kept growing from DIV5 to DIV15. We also counted the number of astrocyte branches that violated tiling. The percentage of astrocytes that expanded over or touched branches from other astrocytes was high on DIV3. However, after DIV5, the percentage of astrocytic branches that violated the tiling rule remained less than 5% while expanding their territories. These results indicate that astrocytes form additional branches and expand to areas not covered by other astrocytes yet.

We analyzed the complexity of astrocyte branches using the topological branch order method (Figure 2c). The topological branch order method counts how many times processes branch, starting from the cell body before reaching their tips. After astrocytes started to branch at DIV5, their branches increased in complexity until DIV15. By DIV15, some astrocyte processes had branched more than 20 times. 

**Figure 3:**
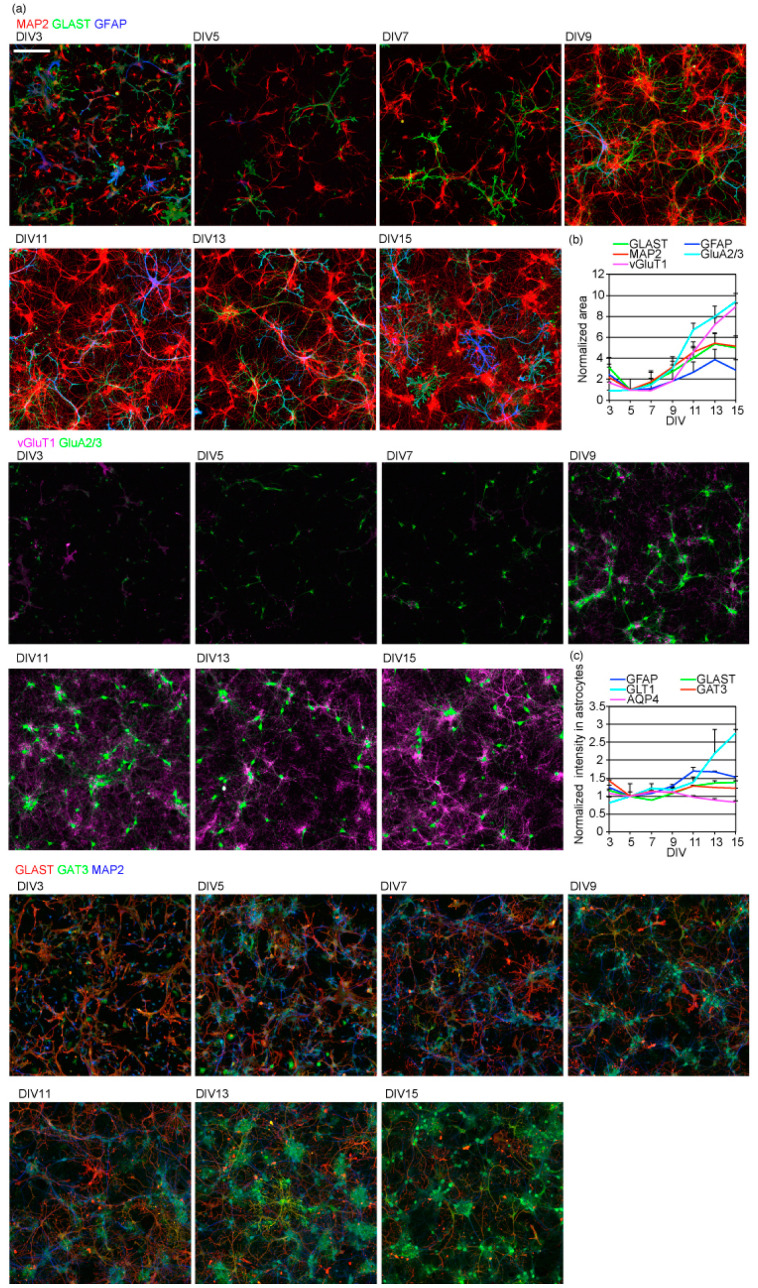
Time-course of astrocytic and neuronal morphological and functional maturation. (**a**) The time-course of neuronal dendrite growth and astrocytic branching and protein expression. Top, MAP2 (red), GLAST (green), GFAP (blue); middle, GluA2/3 (green), vGluT1(magenta); bottom, GLAST (red), GAT3(green), GLT1 (blue). Scale bar, 200 µm. Similar results were obtained for 3 independently prepared cultures. Images of each channel are provided in Figure S3. (**b**) The time-course of neuronal or astrocytic marker positive area. The sizes of each neuronal or astrocytic marker area, with fluorescence signal intensity above the threshold. The values of each marker protein are normalized to that on DIV5. Similar results were obtained for 3 independently prepared cultures. (**c**) The time-course of astrocytic marker expression calculated as the average signal intensity of each marker protein on astrocytes. Each astrocytic marker’s average fluorescence signal intensity above the threshold was calculated. The average fluorescence intensities of each astrocytic marker were normalized to that on DIV5. Similar results were obtained for 3 independently prepared cultures. See Figure 2a for AQP4 images.

Next, we studied how neuronal growth and synaptic maturation relates to astrocyte branching and tiling. At DIV3, neurons had short MAP2 positive dendrites, but astrocytes had not branched and tiled yet (Figure 3a). When neuronal MAP2 positive dendrites started to grow and expand on DIV5, astrocytes also started to branch and tile (Figure 3b). Expression of AMPA-type glutamate receptor GluA2/3 was low at DIV5 and kept increasing to DIV15 (Figure 3b). The signal intensity of presynaptic vesicular glutamate transporter vGluT1 also remained low until DIV7 and increased on DIV9, indicating glutamatergic synapse formation. These results suggest that astrocyte branching and tiling were induced by neurons with their dendrites just starting to grow. Their synapse formation was not required.

We also studied whether astrocytic transporter proteins are induced during morphological maturation. The expression of glutamate transporter GLT1, which is known to be induced through interaction with neurons [2,9], increased most extensively (Figure 3c). The expression of intermediate filament GFAP, another glutamate transporter GLAST, and GABA transporter GAT3 slightly increased. The expression of water channel AQP4 slightly decreased.

The expression levels of GLT1, GLAST, GFAP, AQP4, and GAT3 were heterogeneous among astrocytes prepared from the entire cerebral cortex. The heterogeneity is expected considering the variation in the promoter activity of GLAST and GLT1 depending upon the layers and regions of the cerebral cortex [18]. These proteins’ mean immunofluorescence signal intensities on an astrocyte plasma membrane showed a weak correlation between GLAST, GLT1, GAT3, and AQP4, with their correlation coefficients below 0.5 (Table 1).

### 2.3. Astrocytes Expand Processes to the Area without Other Astrocytes

**Figure 4:**
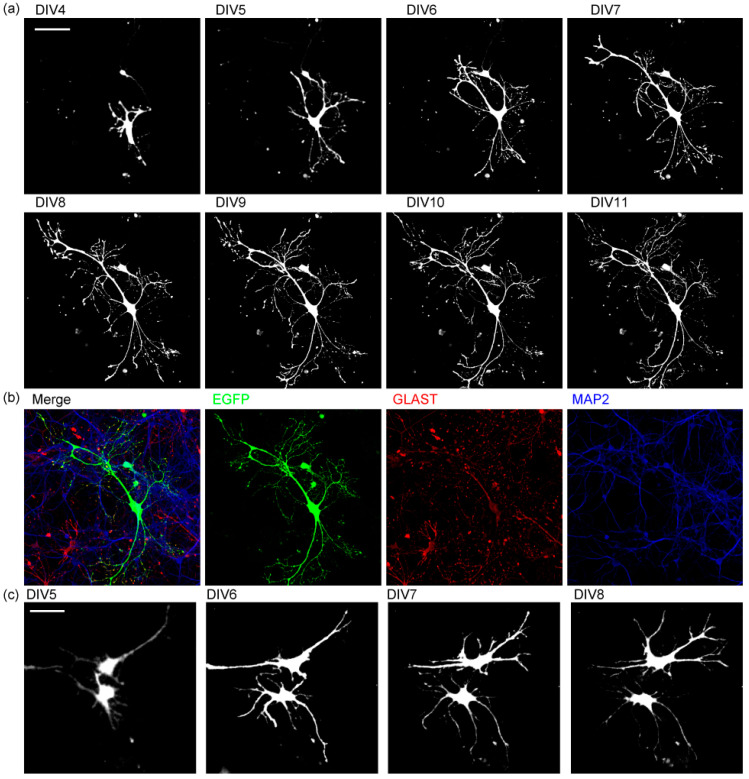
The time-lapse images of astrocyte branch formation. (**a**) The time-lapse images of morphological changes in astrocytes expressing EGFP. *n* = 10. Scale bar, 100 µm. Similar results were obtained for 3 independently prepared cultures with more than 10 cells observed each time. (**b**) Immunostaining of the astrocyte in a. EGFP (green), GLAST (red), MAP2 (blue). (**c**) The time-lapse images of branch formation of two astrocytes expressing EGFP. Scale bar, 50 µm. Similar results were obtained for 3 independently prepared cultures with more than 4 pairs of cells observed each time. See also Video S1.

Next, we took time-lapse images to track the branch formation process of EGFP-labeled astrocytes (Figure 4). Transfection of pEGFP-N1 on DIV2 allowed the expression of EGFP in astrocytes (Figure 4a, Video S1), confirmed by immunostaining of astrocyte marker protein, GLAST (Figure 4b). Neuronal dendrites labeled by MAP2 grew normally. They start to form multi-ordered branches on DIV5. Branches around the cell body were relatively stable. New branches are formed at the edge of the astrocyte territories, or short branches are added inside the territories to make more complex structures. Branches mostly grow in areas not occupied by other branches and rarely expand over other branches. When some branches happened to grow over other branches, they kept expanding to new regions, still mostly avoiding other branches. Even if they grew over other branches, these branches did not retract or were not pruned.

### 2.4. Requirements for Astrocyte Tiling 

**Figure 5:**
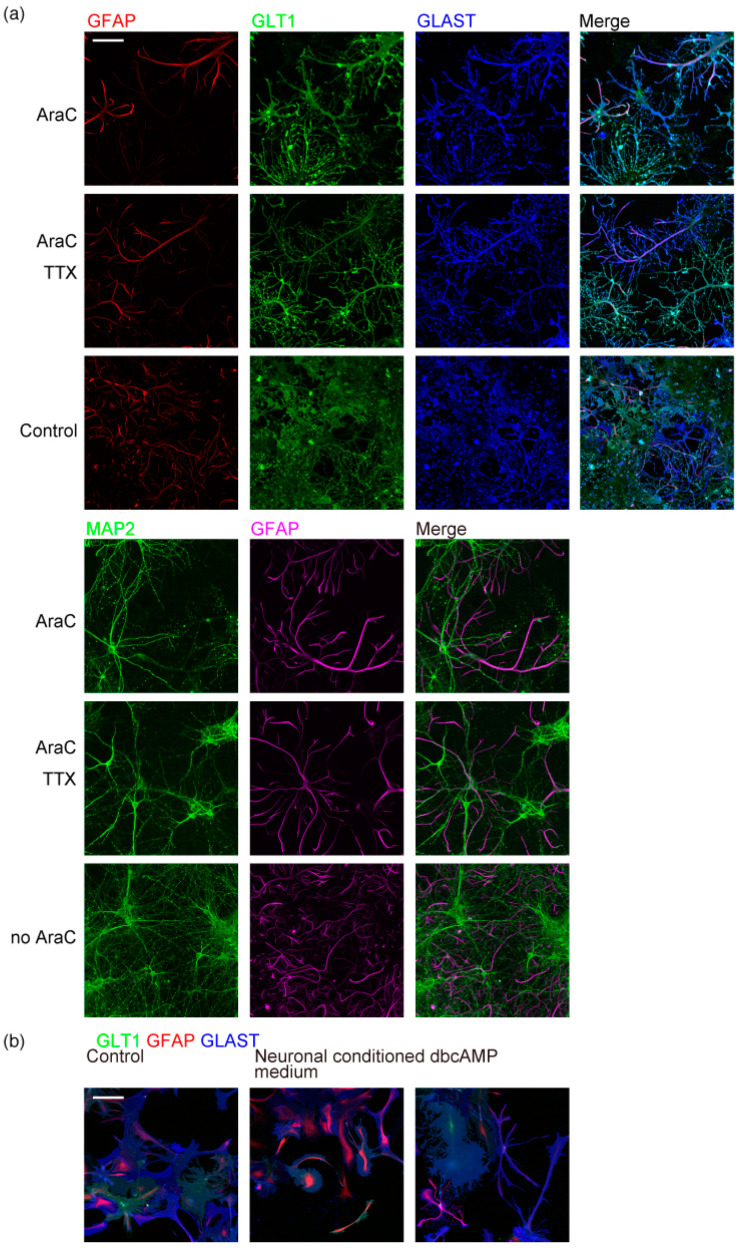
Culture requirements for astrocyte tiling. (**a**) Neuron and astrocytes in rat cerebrum neuron–astrocyte co-culture immunostained on DIV15. *n* = 4. Top, cellular proliferation was suppressed by 5 µM AraC. Middle, proliferation was suppressed by 5 µM AraC, and action potential generation was suppressed by 10 µM TTX supplemented every two days. Bottom, without proliferation suppression. Top, GFAP (red), GLT1 (green), and GLAST (blue). Bottom, MAP2 (green), and GFAP (magenta). Scale bar, 100 µm. Similar results were obtained for 3 independently prepared cultures. (**b**) Astrocytes in rat cerebrum primary glial culture treated with glutamate receptor ligands for 24 h, immunostained on DIV17. GLT1 (green), GFAP (red), and GLAST (blue). Neuronal conditioned medium was obtained from neuron–astrocyte co-culture prepared in parallel. Cells were prepared in parallel with Figure 1a top and imaged under the same conditions with the same linear signal enhancement. Scale bar, 100 µm. Similar results were obtained for 3 independently prepared cultures. Images of each channel are provided in Figure S4.

Next, we studied the requirements of culture conditions for astrocyte tiling. As reported for astrocyte branching [4], suppressing neuronal activity by TTX (tetrodotoxin) every two days did not affect the astrocyte tiling (Figure 5a). This result shows that neuronal action potential is not required for astrocyte tiling. When the astrocyte proliferation was not restricted by AraC, astrocytes formed multi-order branches but did not tile due to high cell density (Figure 5a).

To study whether astrocytes must contact neurons to branch and tile, or substances secreted from neurons are sufficient, we cultured astrocytes in glial culture with the neuronal conditioned medium freshly obtained from neuron–astrocyte co-culture prepared in parallel. Astrocyte monoculture incubated with neuronal conditioned medium for up to 5 days did not show any multi-order branches. Dibutyl cyclic AMP (dbcAMP) is known to cause astrocyte stellation [19,20,21,22]. The application of dbcAMP induced astrocyte stellation but failed to generate multi-order branches (Figure 5b). These results indicate that co-culture with neurons is critical for astrocyte multi-order branching and tiling.

### 2.5. Glutamate Excitotoxicity Damages Neurons and Causes Astrocyte Deformation

**Figure 6:**
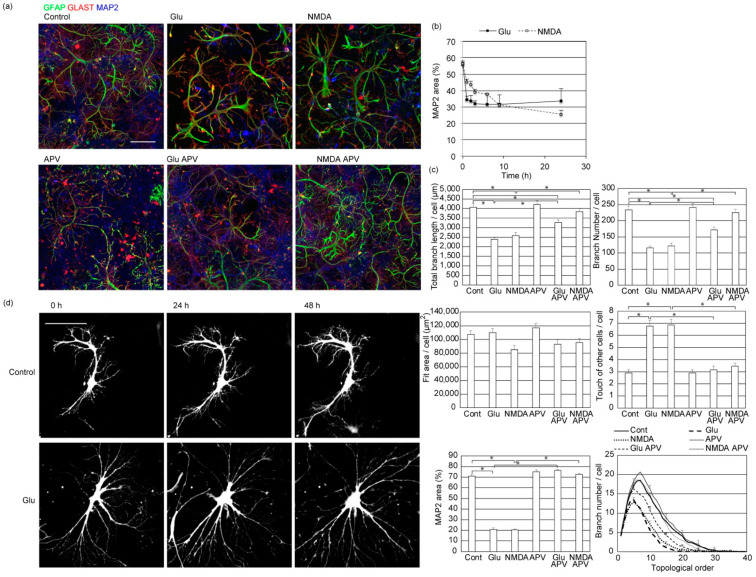
Neuronal damage and astrocyte deformation caused by glutamate. (**a**) Effect of incubation with glutamate receptor ligands for 24 h on rat cerebrum neuron–astrocyte co-culture. Immunostained by astrocytic markers of GFAP (green), GLAST (red), and MAP2 (blue). Similar results were obtained for 3 independently prepared cultures. Scale bar, 100 µm. Images of each channel are provided in Figure S5. (**b**) The time-course of the area covered by neurons. The percentages of neuronal dendritic marker MAP2 positive area were calculated by image thresholding. Mean ± s.e.m. of 8 images. Similar results were obtained for 3 independently prepared cultures. (**c**) Top left, the total length of branches of each astrocyte. Top right, the total number of branches of each astrocyte. Middle left, the area covered by each astrocyte, estimated by the area of a rectangle that covers all branches of each astrocyte. Middle right, the average number of branches that touch or override branches of other astrocytes to violate the tiling rule. Bottom left, the percentages of coverage area by neuronal dendritic marker MAP2. Bottom right, topological branch order analysis for astrocytes. Error bars are shown for every 5 branch orders. Cont-Glu, Cont-NMDA, Cont-GluAPV, NMDA-NMDAAPV, Glu-NMDAAPV pairs were *p* < 0.05 by Tukey–Kramer test of order 10 branch numbers. Mean ± s.e.m. of 92–104 cells. Asterisks, *p* > 0.05 with Tukey–Kramer test. Similar results were obtained for 3 independently prepared cultures. (**d**) The time-course of morphological changes in astrocytes expressing EGFP after application of 20 µM glutamate. Scale bar, 100 µm. Similar results were obtained for 3 independently prepared cultures with more than 10 cells observed each time.

Compared to normal astrocytes organized in non-overlapping spatial domains, reactive astrocytes generated under pathological seizure conditions lose exclusive territories [14]. Reactive astrocytes have thicker and straighter primary branches and fewer process tips. Whether glutamate excitotoxicity is sufficient for tiling violation or other factors such as blood cells or microglia are necessary was not known. To clarify this point, we applied 20 µM glutamate (Figure 6a) to our culture. MAP2 signals of neuronal dendrites were lost within 1 hr after the glutamate application showing neuronal damage (Figure 6b). Under this condition, astrocytes’ branch number and the total length of branches also decreased. The topological analysis of the branch structure showed that astrocyte branches lost complexity. However, the size of the areas covered by an astrocyte did not significantly change. In addition, the number of branches that violated the tiling rule and overrode other astrocyte processes increased. These results indicate that glutamate application damaged neurons and reduced the number of astrocyte processes and their tiling, while the size of the area they covered was not affected.

NMDA (N-methyl-D-aspartate)-type glutamate receptors are calcium-permeable, and their excess activation is the primary cause of neuronal damage due to excitotoxicity. Accordingly, 100 µM APV (2-amino-5-phosphonopentanoic acid), the NMDA receptor antagonist, completely blocked the loss of MAP2 positive neuronal dendrites induced by 20 µM glutamate (Figure 6a,b). Under this condition, astrocyte processes and their tiling were not affected. Glutamate also activates other ionotropic and metabotropic glutamate receptors and affects astrocytes’ energy and ion balance through action on glutamate transporters. Therefore, we studied the effect of 20 µM NMDA application. This also caused neuronal damage, retraction of astrocyte processes, and tiling loss. Again, 100 µM APV blocked both neuronal damage and astrocyte deformation caused by 20 µM NMDA. These results suggest that neurons are required to maintain tiling and the process tips of astrocytes. However, we cannot exclude the possibility that glutamate or NMDA acting directly on astrocytes affect their morphology.

We also applied glutamate to EGFP-labeled astrocytes to track astrocyte process movement. Astrocyte processes in the presence of neurons tend to be stable once formed, and they further expand new processes from there. After glutamate application, these astrocytes rapidly changed their morphology and explored the area (Figure 6d, Video S2). Astrocyte processes dynamically expanded and retracted in the absence of neurons, while astrocytes with neurons did not grow in other astrocytes’ directions. The movements and exploration show how astrocyte tiling is lost after excitotoxic glutamate application. 

## 3. Discussion

This study showed how astrocytes tile when cultured with neurons. The previous literature shows astrocyte branches in the presence of neurons [2,4,6,7,19]. In addition, three-dimensional cultures are used to reproduce astrocyte morphology and properties in vitro [23,24]. However, it is hard to maintain astrocytes that tile and form multi-ordered branches. Our neuron–astrocyte co-culture in a regular cell culture plate accomplished this by using an astrocyte conditioned medium to minimize the perturbation.

We used the entire cerebral cortex to prepare the culture. Most cells formed multi-ordered branches indicating that astrocytes in the cerebellar cortex can branch and tile under this condition. Using this culture, we could show that immature neurons before synapse formation that had just started neurite extension induce astrocyte’s branching and tiling. Astrocytes functionally mature while forming branches. The expression of glutamate or GABA transporters, which are required to support and regulate synaptic transmission, increased. During brain development, astrocytes form territories, but borders between them are completed within a month after the birth of rats [13]. Astrocytes in our study expanded their branches while avoiding the direction already occupied by other astrocytes from the beginning. The mechanism of astrocyte tiling and boundary formation could be different. Spongiform processes may be necessary for clear boundary formation. It is reasonable for astrocytes to expand branches while avoiding areas already covered by other astrocyte branches. This way, astrocytes uniformly cover the brain to prepare for neurotransmitter uptake and avoid neuronal death due to excitotoxicity before synapse formation.

There are several ways how cells with multiple processes tile. Thousands of spongiform processes of an astrocyte may avoid the entry of other astrocyte processes sterically. However, astrocytes are finely branched but not to the extent of preventing access to other processes in our culture. Processes of astrocytes cultured without the restriction of proliferation override other astrocyte processes, indicating that steric hindrance of process penetration does not play a role in tiling. Astrocytes in a healthy brain will receive clues to restrict proliferation to keep the population of astrocytes adequate. Another possibility is pruning or retracting processes that override other astrocytes. Our time-lapse imaging of growing astrocyte processes showed that once a process has overridden other processes, it keeps growing without retraction or pruning and expands new tiled territories in the area they had grown. These observations indicate that astrocytes tile by contact- or proximity-mediated repulsion and not by pruning.

Molecules responsible for astrocyte tiling are not known. If the tiling mediators stop branch expansion around other astrocyte branches, the strict discrimination of self and non-self is not necessary. The molecule inducing the repulsion could be some diffusible mediator or extracellular matrix components that specifically affect astrocyte process growth. The tiling mediator should be astrocyte-specific because astrocytes do not avoid neurons, and neurons we prepared from the cerebral cortex did not tile or avoid each other.

Some neuronal molecules are responsible for contact-dependent repulsive interactions between neighboring neurons of the same type. Several neurons are known to tile, such as leech mechanosensory neurons [25,26], dendritic arborization (da) sensory neurons of *Drosophila* [27,28,29,30], and rat retinal ganglion cells [31,32]. Single-pass transmembrane proteins with structural diversity and homophilic interaction, such as Dscams [33,34,35,36,37] and protocadherins [38,39,40], contribute to neuronal self- and non-self-discrimination. Moreover, these molecules interact with other repulsive guidance molecules such as Slit-Robo and netrin [41,42,43,44]. These, or molecules with similar functions, could be responsible for astrocyte tiling.

The interaction between astrocytic neuroligins and neuronal neurexins mediates astrocyte branching [45]. Although neurons induce astrocyte branching and tiling, neurons are not absolutely required for astrocyte branching, because astrocytes isolated from the developed brain by immunopanning form multi-order branches [46]. In our study, neurons are necessary to maintain the tiling, and the neuronal conditioned medium was insufficient. Some short-lived or contact-dependent neuronal factors may be necessary for astrocyte tiling.

When astrocytes are released from neurons after neuronal damage, astrocytes start exploring other neurons to interact with or phagocytose cellular debris if necessary. Astrocytes lost some branches but kept multi-order branches after the neuronal loss induced by glutamate and did not take a polygonal form observed in astrocyte monoculture. The change in astrocyte morphology and dynamics may help them protect neurons and remove their debris.

Our time-course study of astrocyte branch formation and tiling showed that neurons induce astrocytes to form branches that avoid other branches at an early stage of neurite expansion. Astrocytes reproducibly branch and tile in our culture, and we can use this culture to screen gene products responsible for interaction with neurons, branching, and tiling in the future.

## 4. Materials and Methods

### 4.1. Materials

Primary antibodies used for immunostaining were Guinea pig polyclonal anti-GLT1 (GLT1-GP-Af810, Frontier Institute, Hokkaido, Japan, 1:2000 dilution), rabbit polyclonal anti-GLAST (GLAST-Rb-Af660, Frontier Institute, 1:2000 dilution), mouse monoclonal anti-glial fibrillary acidic protein (GFAP) GA5 (GTX73615, Genetex, Irvine, CA, USA, 1:2000 dilution), Guinea pig polyclonal anti-GABA transporter 3 (GAT3) (274 304, Synaptic Systems, Göttingen, Germany, 1:1000 dilution), rabbit polyclonal anti-aquaporin 4 (AQP4) (sc-20812, Santa-Cruz Biotechnologies, Dallas, TX, USA, 1:200 dilution), rabbit monoclonal phospho-Ezrin (Thr567)/Radixin (Thr564)/Moesin (Thr558) (48G2) (#3726, Cell Signaling Technology, Danvers, MA, USA), chicken polyclonal anti- microtubule-associated protein MAP2 (RRID:AB_2138153, Abcam, Cambridge, UK, 1:5000 dilution), rabbit polyclonal anti-GluA2/3 (RRID:AB_90710, Merck Millipore, Burlington, VT, USA, 1:1000 dilution), mouse monoclonal anti-vGluT1 (RRID: AB_2750766, UC Davis/NIH NeuroMab Facility, Davis, CA, USA, 1:1000 dilution), anti-AQP4 antibody E5415A [47] (1:10 dilution of culture supernatant, RCB4883, RIKEN BRC, Ibaraki, Japan). For secondary antibodies, Alexa 488 anti-chicken (A21449), Alexa 488 anti-rabbit (A11008), Alexa 488 anti-Guinea pig (A11073), Alexa 555 anti-Guinea pig (A21435), Alexa 647 anti-rabbit (A32733), Alexa 647 anti-rabbit (A32933), and Alexa 647 anti-mouse (A11001) antibodies are all from Thermo Fisher Scientific, Waltham, MA, USA and used at 1:3000 dilution.

### 4.2. Animals

The animal study protocol was approved by the Institutional Review Board of the Animal Research Committee of the Showa Women’s University (21-05 23 April 2021) and the International University of Health and Welfare (16131NA 30 June 2016, 18025NA 28 March 2019), and conformed to the relevant regulatory standards. We treated all animals according to Japanese national guidelines and regulations, following the Guide for the Care and Use of Laboratory Animals. Sprague-Dawley rats (RRID: RGD_10395233, Japan SLC, Shizuoka, Japan) were sacrificed for culture preparation under sevoflurane anesthesia on arrival from the supplier.

### 4.3. Statistical Analysis

Statistical analysis was performed after normality assessment using a one-way analysis of variance combined with Tukey’s or Tukey–Kramer’s test in R (R Foundation for Statistical Computing, Vienna, Austria). Correlation coefficients of astrocyte transporters’ expression levels are calculated using Microsoft Excel 2019.

### 4.4. Primary Neuron–Astrocyte Co-Culture and Glial Culture

Before starting the culture preparation, 96-well cell culture plates (TR5003, Trueline, La Crosse, WI, USA) or 8-well glass slide chambers (154534, Thermo Fisher Scientific, Waltham, MA, USA) were pre-coated with 0.1% poly-l-lysine in 0.1 M Na-Borate buffer (pH 8.5) for more than 12 h at 25 °C. After coating, we washed the plates with sterile phosphate-buffered saline three times. Then, we added 50 µL (96-well plate) or 150 µL (8-well slide chamber) of astrocytic conditioned medium described below to each well and kept cultures at 37 °C in a humidified atmosphere containing 50 mL/L CO_2_.

Ten brains of 1-day-old Sprague-Dawley rats of both sexes were aseptically removed and dipped into ice-cold Gey’s balanced salt solution (0.22 g/L CaCl_2_·2H_2_O, 0.37 g/L KCl, 0.03 g/L KH_2_PO_4_, 0.21 g/L MgCl_2_·6H_2_O, 0.07 g/L MgSO_4_·7H_2_O, 8.0 g/L NaCl, 0.227 g/L NaHCO_4_, 0.12 g/L Na_2_HPO_4_, 1.0 g/L D-glucose) in a petri dish bubbled with 50 mL/L CO_2_, 950 mL/L O_2_ mixture. We transferred the entire cerebral cortex to a new petri dish with bubbled ice-cold Gey’s balanced salt solution and removed dura matter by forceps. After that, the cortexes were transferred to a glass petri dish and dissected into <1 mm pieces with scalpels. The dissected cortexes were suspended in 12 mL of 0.25 *w*/*v*% Trypsin, 1 mmol/L EDTA-4Na (209-16941, Fujifilm Wako, Osaka, Japan) supplemented with 1 mg/mL deoxyribonuclease I from bovine pancreas (043-26773, Fujifilm Wako, Osaka, Japan), and 0.75% sucrose. The tissues were dissociated by pipetting. The dissociated tissues were incubated at 37 °C for 10 min with occasional gentle swirling. We added 9 mL of FBS (fetal bovine serum, S1820, Biowest, Nuaillé, France) to stop the digestion and gently mixed it by pipetting. The dissociated cells were centrifuged at 400× *g* for 5 min. The pelleted cells were suspended in 4 mL of MACS Neuro Medium (130-093-570, Miltenyi Biotec, Bergisch Gladbach, Germany) supplemented with 2% MACS NeuroBrew-21 supplement (130-093-566, Miltenyi Biotec, Bergisch Gladbach, Germany), 0.4 mM Glutamine, and 0.1% antibiotic-antimycotic (15240032, Thermo Fisher Scientific, Waltham, MA, USA) (NM-NB21). The tissue aggregates were removed by filtration through a 25 mm filter holder (SX0002500, Merck, Darmstadt, Germany) with three layers of lens cleaning tissue (2105-802, GE Healthcare, Chicago, IL, USA). Cells were diluted with NM-NB21 to 60,000 cells/mL density, corresponding to 50–60 mL after dilution. The cell suspensions of 50 µL (96-well plate) or 150 µL (8-well slide chamber) were plated to each well and cultured at 37 °C in a humidified atmosphere containing 50 mL/L CO_2_. It takes approximately two hours from brain removal to plating. Half of the culture medium was replaced with NM-NB21 supplemented with 10 µM of AraC to the final concentration of 5 µM AraC on DIV1 unless otherwise described. The primary neuron–astrocyte co-culture should be kept without medium change afterward. Where indicated, TTX to the final concentration of 10 µM was added on DIV1 and further added to 10 µM for every following two days.

Primary glial cultures were obtained by further diluting the cell suspension 10-fold with DMEM (Dulbecco modified Eagle medium, 043-30085, Fujifilm Wako, Osaka, Japan) supplemented with 10% FBS and 1% penicillin-streptomycin solution (168-23191, Fujifilm Wako), and plating to non-coated 75 cm^2^ culture flask (TR6002, Trueline, La Crosse, WI, USA). The culture medium of the primary glial cultures was changed every three days until DIV15, and microglia were removed by shaking at 180 rpm, 37 °C for 24 h. Then, the primary glial cultures were incubated with DMEM supplemented with 5% FBS and 1% penicillin-streptomycin. The conditioned medium was harvested every three days until DIV35, pooled, sterile-filtrated, and used as the astrocytic conditioned medium for the neuron–astrocyte co-culture.

### 4.5. Fluorescent Protein Expression

pEGFP-N1 (6085-1, Clontech, now Takara Bio Inc., Shiga, Japan) was introduced to astrocytes using Lipofectamine LTX and PLUS (15338100, Thermo Fisher Scientific, Waltham, MA, USA) on DIV2. For each well of a rat cerebrum neuron–astrocyte co-culture in 96-well plates, 0.15 µg plasmid, 0.15 µL Lipofectamine LTX, and 0.05 µL PLUS in 5 µL Opti-MEM (31985062, Thermo Fisher Scientific, Waltham, MA, USA) were used. Fluorescence signals were observed within 8 h after transfection and lasted for ten days.

### 4.6. Microscopy Imaging 

Live and fixed images were obtained using a CQ1 Benchtop High-Content Analysis System (Yokogawa Electric Corporation, Tokyo, Japan) with the UPLSAP010x2 lens (Olympus, Tokyo, Japan) with 0.4 numerical aperture. The excitation wavelength was 488 nm for EGFP and Alexa 488, 561 nm for Alexa 555, and 640 nm for Alexa 647. In addition, A1 plus laser scanning confocal microscope system with a Plan Apo VC 20× DIC N2 lens with a numerical aperture of 0.75 was also used to take fixed images using NIS Elements software (Nikon, Tokyo, Japan). Excitation wavelengths were 488 nm for Alexa 488, 561 nm for Alexa 555, and 638 nm for Alexa 647 secondary antibodies.

### 4.7. Morphology Analysis

ImageJ [48]-based FIJI [49] software was used for morphological analysis. For branch analysis, fluorescence images of astrocytes immunostained by anti-GFAP are binarized and then skeletonized [50]. The obtained skeleton structures were manually edited, referring to the original anti-GFAP, anti-GLAST, and anti-GLT1 stained images. Branch length, location, total number, and length of branches were calculated by the “analyze skeleton” plugin of ImageJ [50]. Topological branch order analysis was performed by manually written Microsoft Excel macro run for the “analyze skeleton” output. We estimated the area of coverage by the astrocytes using the “bound rectangle” measurement function of FIJI, applied to the region of interest assigned to each skeletonized astrocyte.

## Figures and Tables

**Table 1:** The correlation between a pair of transporter protein expression levels for each astrocyte. Immunofluorescence signal intensities of each transporter protein for each astrocyte were measured as average signal intensity on the astrocyte cell body. The correlation coefficients between transporter protein signal intensities for all astrocytes in each image were calculated. Average correlation coefficients of independently prepared samples (*n*), with 65–126 cells for each analysis.

	Correlation Coefficient (Average)	*n*
GLAST–GLT1	0.23	10
GLAST–GAT3	0.46	5
GLAST–AQP4	0.27	5
GLT1–GAT3	0.46	5
GLT1–AQP4	0.39	5

## Data Availability

Not applicable.

## Supplementary Materials

The following supporting information can be downloaded at: https://www.mdpi.com/article/10.3390/ijms23084161/s1.
