# Supplementary figures and images for: Neurons Induce Tiled Astrocytes with Branches That Avoid Each Other
Source: Int J Mol Sci. 2022 Apr 9;23(8):4161. doi: 10.3390/ijms23084161 (PMC9028504; doi:10.3390/ijms23084161)

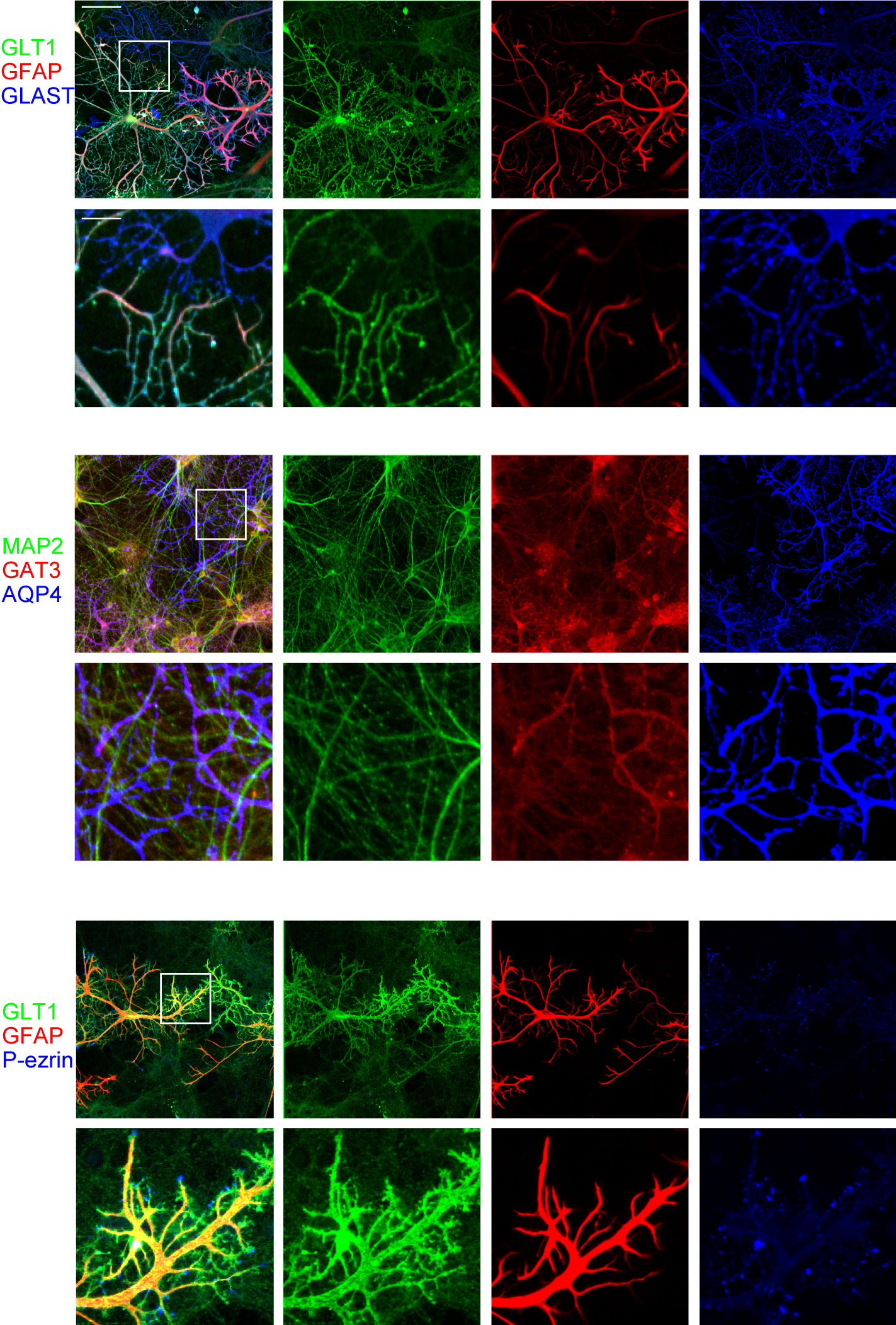

Supplement: Supplementary file 1 [file ijms-23-04161-s001.zip › FigS1.jpg]

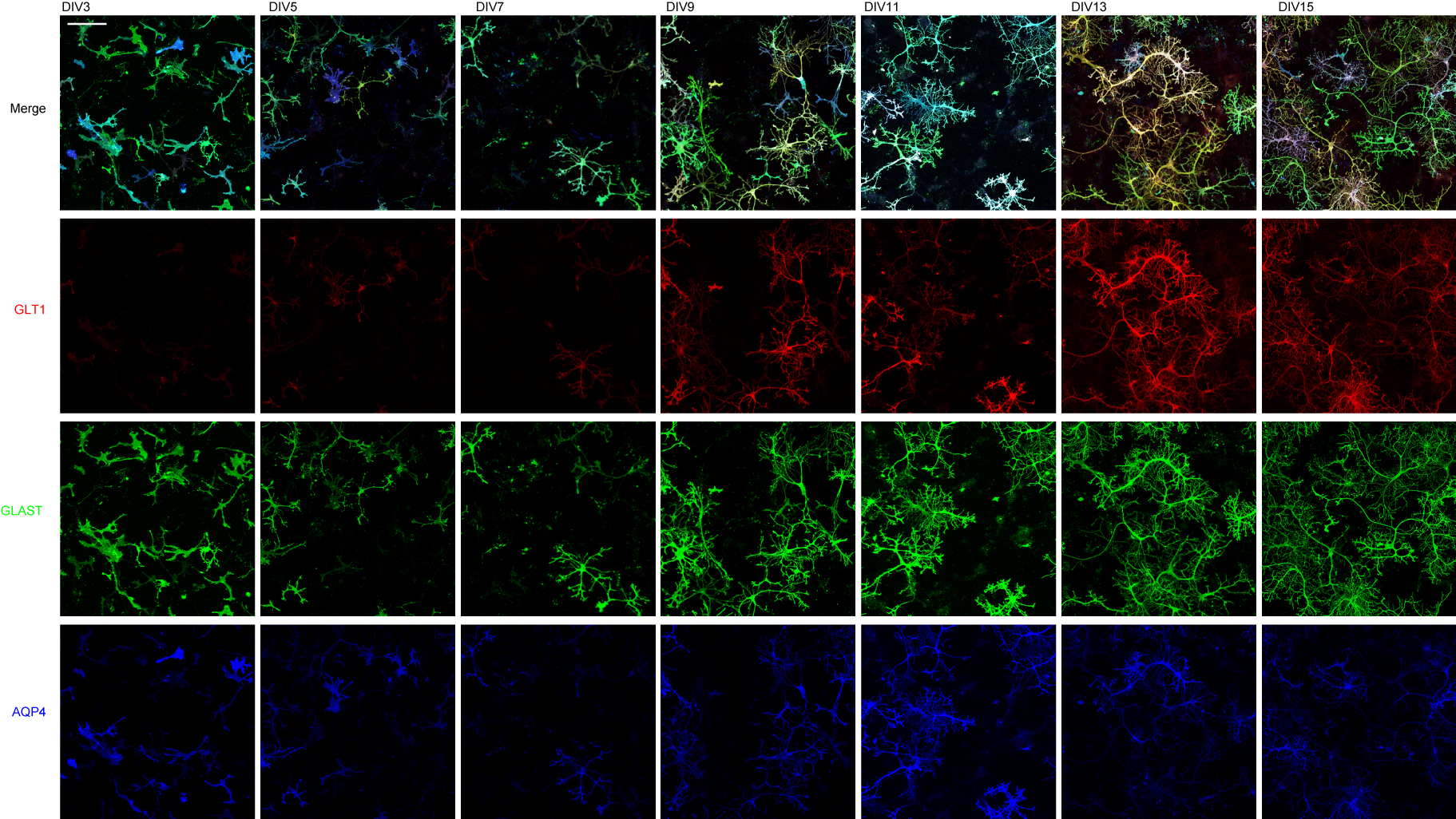

Supplement: Supplementary file 1 [file ijms-23-04161-s001.zip › FigS2.jpg]

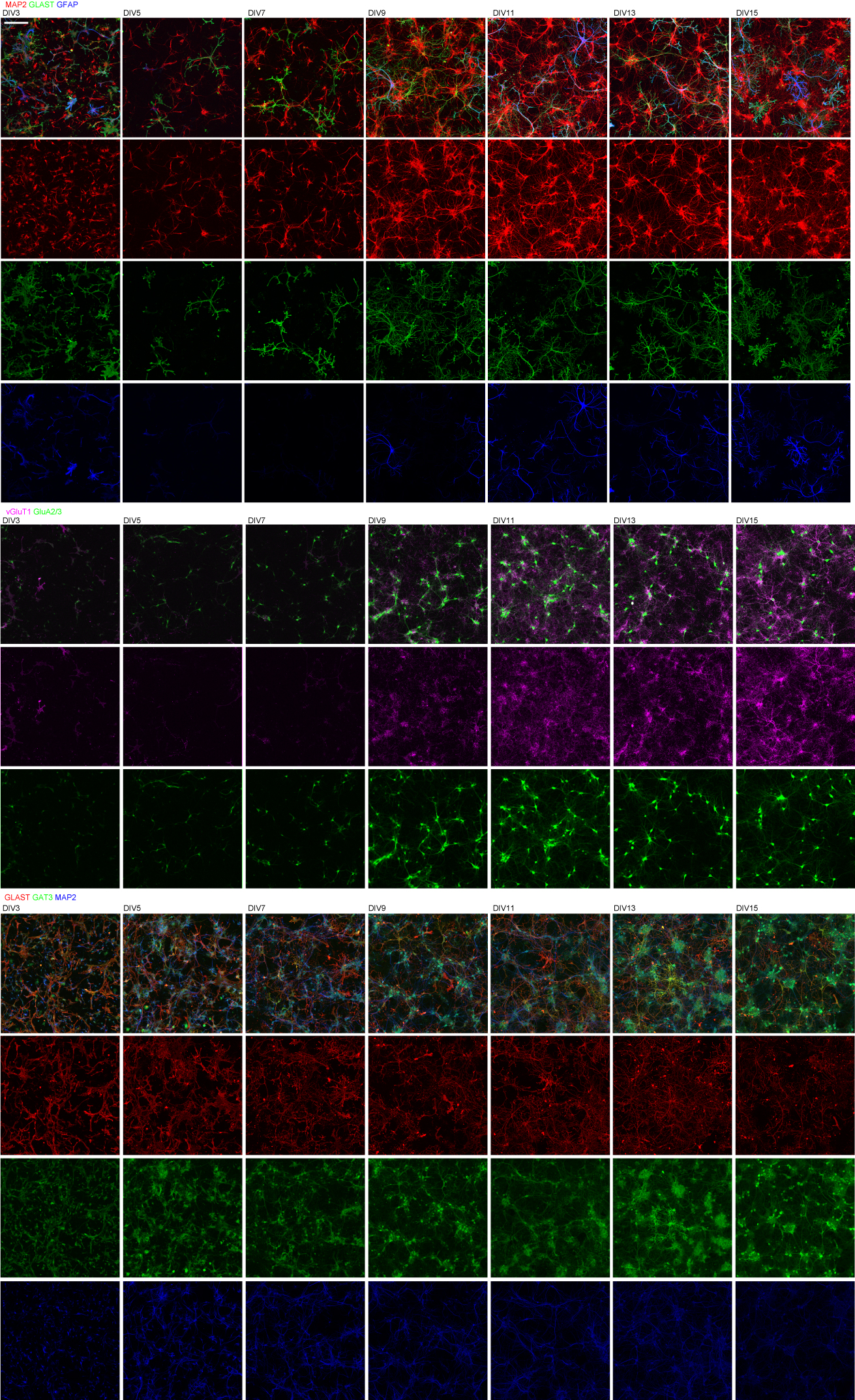

Supplement: Supplementary file 1 [file ijms-23-04161-s001.zip › FigS3.jpg]

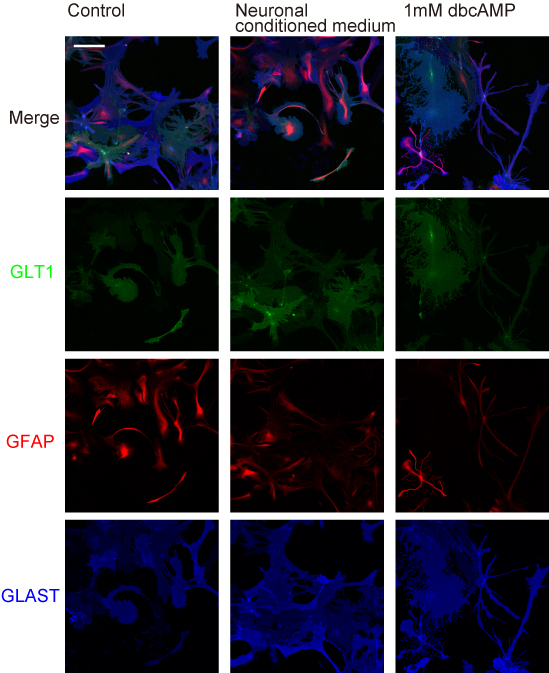

Supplement: Supplementary file 1 [file ijms-23-04161-s001.zip › FigS4.jpg]

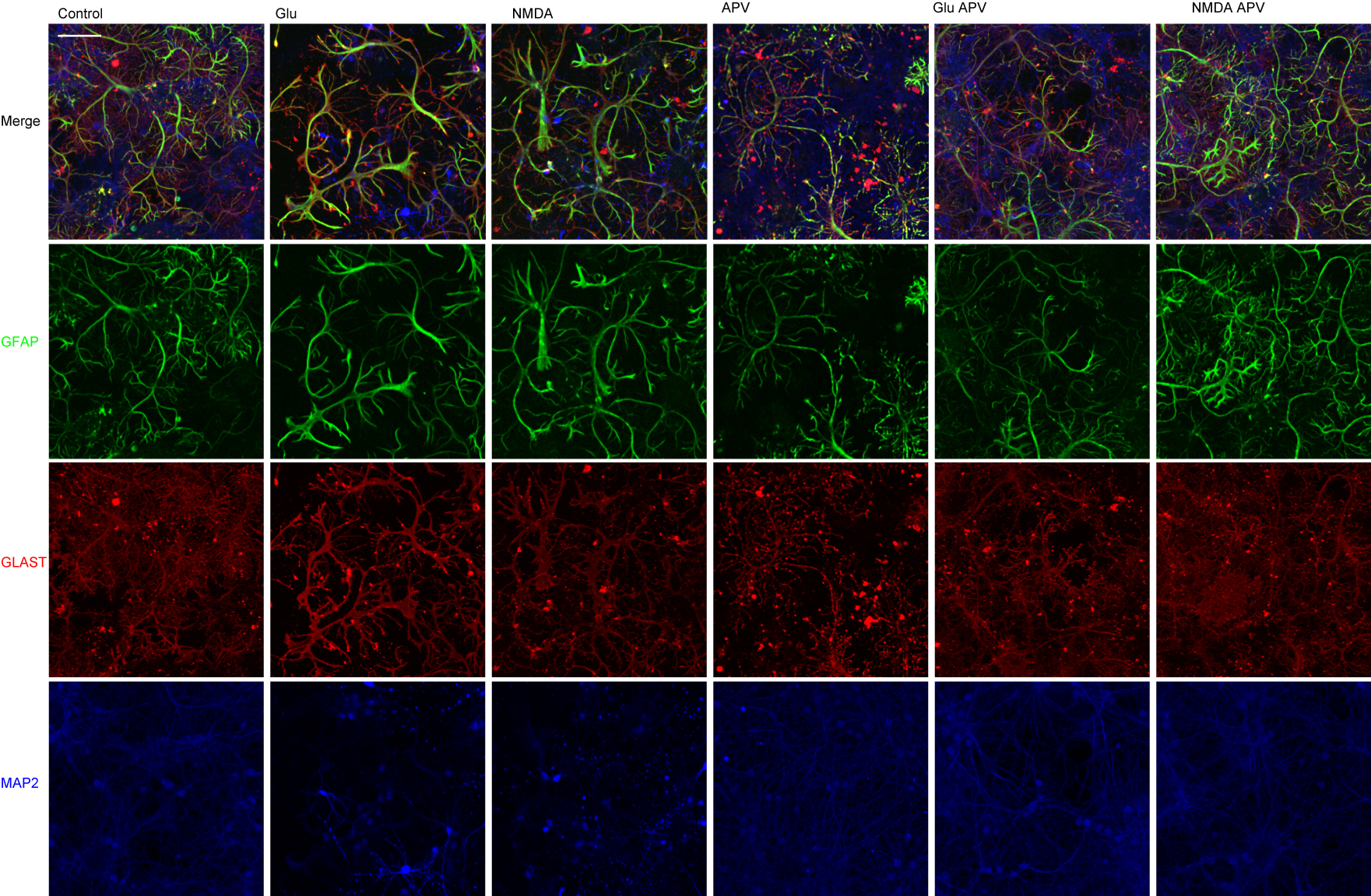

Supplement: Supplementary file 1 [file ijms-23-04161-s001.zip › FigS5.jpg]
